# Pathophysiology of diabetic dyslipidaemia: where are we?

**DOI:** 10.1007/s00125-015-3525-8

**Published:** 2015-03-01

**Authors:** Bruno Vergès

**Affiliations:** 1 Service Endocrinologie, Diabétologie et Maladies Métaboliques, Hôpital du Bocage, 2 bd Maréchal de Lattre de Tassigny, 21000 Dijon, France; 2 INSERM CRI 866, Medicine University, Dijon, France

**Keywords:** Cardiovascular disease, Dyslipidaemia, HDL-cholesterol (HDL-C), Insulin resistance, LDL-cholesterol (LDL-C), Lipid metabolism, Review, Triglycerides, Type 2 diabetes mellitus

## Abstract

Cardiovascular disease is a major cause of morbidity and mortality in patients with type 2 diabetes mellitus, with a two- to fourfold increase in cardiovascular disease risk compared with non-diabetic individuals. Abnormalities in lipid metabolism that are observed in the context of type 2 diabetes are among the major factors contributing to an increased cardiovascular risk. Diabetic dyslipidaemia includes not only quantitative lipoprotein abnormalities, but also qualitative and kinetic abnormalities that, together, result in a shift towards a more atherogenic lipid profile. The primary quantitative lipoprotein abnormalities are increased triacylglycerol (triglyceride) levels and decreased HDL-cholesterol levels. Qualitative lipoprotein abnormalities include an increase in large, very low-density lipoprotein subfraction 1 (VLDL_1_) and small, dense LDLs, as well as increased triacylglycerol content of LDL and HDL, glycation of apolipoproteins and increased susceptibility of LDL to oxidation. The main kinetic abnormalities are increased VLDL_1_ production, decreased VLDL catabolism and increased HDL catabolism. In addition, even though LDL-cholesterol levels are typically normal in patients with type 2 diabetes, LDL particles show reduced turnover, which is potentially atherogenic. Although the pathophysiology of diabetic dyslipidaemia is not fully understood, the insulin resistance and relative insulin deficiency observed in patients with type 2 diabetes are likely to contribute to these lipid changes, as insulin plays an important role in regulating lipid metabolism. In addition, some adipocytokines, such as adiponectin or retinol-binding protein 4, may also contribute to the development of dyslipidaemia in patients with type 2 diabetes.

## Introduction

The risk of cardiovascular disease and cardiovascular mortality is significantly increased in patients with type 2 diabetes mellitus relative to healthy individuals [1, 2]. A major contributor to the increased cardiovascular risk associated with type 2 diabetes is dyslipidaemia, which encompasses abnormalities in all lipoproteins [3–5]. Lipid abnormalities observed in type 2 diabetes are not only quantitative, but also qualitative and kinetic in nature [6–8]. A number of factors may contribute to the changes in lipid metabolism in patients with type 2 diabetes, including insulin resistance and/or relative insulin deficiency, adipocytokines (e.g. adiponectin), and hyperglycaemia [6–8]. The aim of this review is to briefly describe normal lipoprotein metabolism, including the role of insulin, to describe the pathophysiology of the lipid abnormalities observed in individuals with type 2 diabetes, and to discuss how these lipid abnormalities relate to the development of cardiovascular disease.

## Overview of normal lipoprotein metabolism

**Fig. 1:**
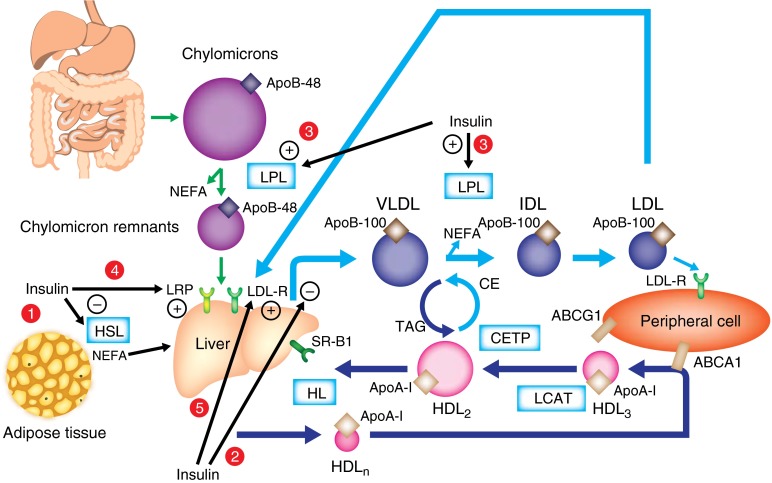
An overview of human lipoprotein metabolism and the effects of insulin on lipoprotein metabolism. (1) Insulin inhibits hormone-sensitive lipase. (2) Insulin inhibits hepatic VLDL production. (3) Insulin activates LPL. (4) Insulin increases LRP expression on the plasma membrane. (5) Insulin increases LDL receptor (LDL-R) expression. CE, cholesterol ester; CETP, cholesteryl ester transfer protein; HDL_n_, nascent HDL HL, hepatic lipase; HSL, hormone-sensitive lipase; LPL, lipoprotein lipase; SR-B1, scavenger receptor B1; TAG, triacylglycerol

Lipids are transported within body fluids in the form of lipoprotein particles, which are classified according to their density, ranging from chylomicrons to VLDL, intermediate-density lipoprotein (IDL), LDL and HDL (Fig. 1).

### Postprandial lipidaemia and chylomicrons

Dietary lipids are absorbed by the enterocytes via passive diffusion or specific transporters (e.g. CD36 for NEFA and Niemann-Pick C1-like 1 protein [NPC1L1] for cholesterol). Within the enterocytes, triacylglycerols (triglycerides), cholesteryl esters and other lipids (phospholipids and small amounts of unesterified cholesterol) are associated with apolipoprotein (Apo)B-48 (as well as ApoA-IV and ApoA-I) to form chylomicrons in a process involving microsomal triacylglycerol transfer protein (MTP) and fatty acid transport proteins. Chylomicrons are then exported into lymph and subsequently into the blood. ApoB-48 synthesis by the gut occurs continuously; however, lipidation to form chylomicrons is dependent on the availability of lipids and occurs mainly after meals.

Lipoprotein lipase (LPL), which is attached to the luminal surface of endothelial cells and present mostly in muscles, the heart and the adipose tissue, plays a major role in chylomicron clearance by hydrolysing triacylglycerols and liberating NEFA into the circulation. The chylomicron remnants produced by the lipolysis of chylomicrons are taken up by the liver via the LDL receptor (Fig. 1) in conjunction with the LDL receptor-related protein (LRP), both of which bind ApoE.

### VLDLs and IDLs

**Fig. 2:**
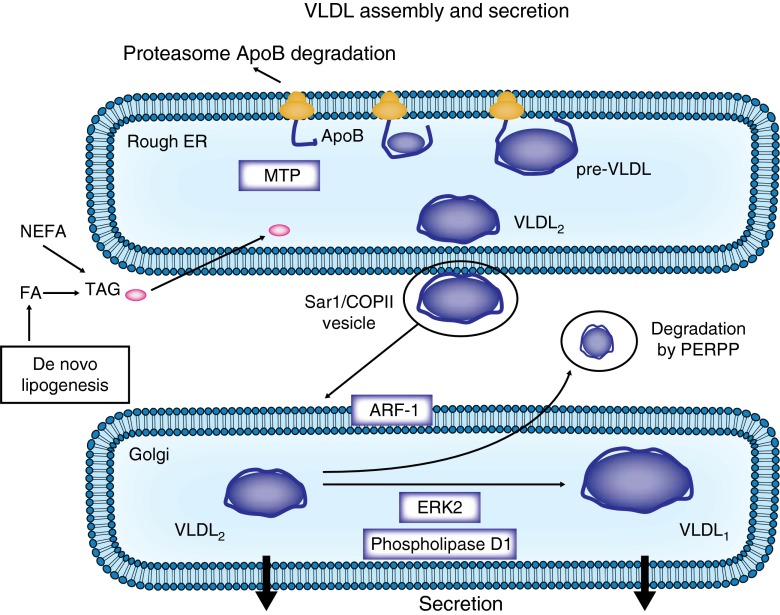
An overview of VLDL assembly and secretion. Step 1: In the rough ER, ApoB is lipidated by MTP, leading to the formation of pre-VLDL, then VLDL_2_ by further lipidation. VLDL_2_ exits the ER compartment via Sar1/COPII vesicles, which are directed to the Golgi apparatus. ARF-1 is involved in VLDL_2_ trafficking between the ER and the Golgi apparatus. Step 2: In the Golgi apparatus, VLDL_2_ is converted into larger VLDL_1_ by the addition of lipids. This step is promoted by phospholipase D1 and extracellular signal-regulated kinase 2 (ERK2). At this stage degradation by PERPP may occur. COPII, coat protein II; FA, fatty acid; MTP, microsomal triglyceride transfer protein; TAG, triacylglycerol

Lipids are exported from the liver into the blood as VLDLs. The first step of VLDL assembly takes place in the rough endoplasmic reticulum (ER) where ApoB-100 is cotranslationally and post-translationally lipidated by MTP, forming pre-VLDL [9, 10]. In the absence of adequate core lipids and/or MTP, partially translocated ApoB is exposed to the cytosol and subjected to degradation via the ubiquitin–proteasome system. During the second step, pre-VLDL is further lipidated late in the ER compartment to form VLDL_2_, exiting the ER compartment via Sar1 (a GTPase)/coat protein II (COPII) vesicles that fuse to the *cis* side of the Golgi apparatus. In the Golgi apparatus, VLDL_2_ can be converted into larger VLDL_1_ by the addition of lipids (Fig. 2). At this stage, VLDL particles may also be degraded via post-ER presecretory proteolysis (PERPP) [11]. The formation of VLDL_1_ depends on factors such as ADP ribosylation factor 1 (ARF-1), phospholipase D1 and extracellular signal-regulated kinase 2 (ERK2), which are involved in membrane trafficking between the ER and the Golgi apparatus or in the formation of cytosolic lipid droplets [10].

As with chylomicrons, triacylglycerols from VLDLs are hydrolysed by LPL in plasma, producing NEFA to be used as fuel in the heart and skeletal muscle or for storage within adipocytes (as triacylglycerols). The progressive triacylglycerol depletion of VLDLs induces the transfer of a portion of the lipoprotein surface layer (including phospholipids, ApoC and ApoE) to HDLs and leads to the formation of IDLs [8]. Approximately 90% of IDLs are converted into LDL through further lipolysis involving hepatic lipase, which has both triacylglycerol lipase and phospholipase activities, whereas the rest is cleared by the liver (via LRP or LDL receptors).

### LDLs

LDL, the major transporter of cholesterol within the blood, comprises a core of esterified cholesterol molecules enclosed in a shell of phospholipids and unesterified cholesterol, together with a single molecule of ApoB-100. LDL is taken up into cells via receptor-mediated endocytosis, which involves, first, the binding of LDL–ApoB-100 to the LDL receptor on the plasma membrane of hepatic and other tissues, then the internalisation of the LDL-receptor complex via endocytosis, followed by fusion with lysozymes, which contain a number of catabolic enzymes. Proprotein convertase subtilisin/kexin type 9 (PCSK9) plays a key role in regulating LDL-receptor activity by binding the LDL-receptor/LDL complex and directing the receptor away from recycling back to the surface and into the lysosomal catabolic pathway.

### HDLs

The atheroprotective effect of HDLs related to their role in reverse cholesterol transport is well known, but these lipoprotein particles also have anti-inflammatory, anti-oxidative, anti-thrombotic and anti-apoptotic properties. HDLs are synthesised by both the liver and the intestine. Newly secreted or nascent HDLs contain only apolipoproteins (mainly ApoA-I) and rapidly recruit non-esterified cholesterol and phospholipids from peripheral cells through the binding of ApoA-I to the membrane-associated ATP-binding cassette protein 1 (ABCA1) transporter, allowing for the transport of non-esterified cholesterol and phospholipids from the cytoplasm into HDL (Fig. 1) [12]. Within the HDL particle, non-esterified cholesterol is esterified by lecithin–cholesterol acyltransferase (LCAT). In the circulation, HDLs acquire more cholesterol from peripheral tissues, including macrophages within artery walls, again through the ABCA1 transporter, as well as the ATP-binding cassette G1 (ABCG1) transporter. During this process, small-size HDL (usually called HDL_3_) becomes larger (usually called HDL_2_). HDL returns cholesterol to the liver via both direct and indirect mechanisms. Through scavenger receptor B1 (SR-B1), HDL-cholesteryl esters are directly taken up by the liver, where they are hydrolysed. HDL also exchanges lipids with VLDL and LDL in a process involving cholesteryl ester transfer protein (CETP), whereby cholesteryl esters are transferred from HDL to VLDL and, reciprocally, triacylglycerols are transferred from VLDL to HDL. In the circulation, HDL also receives ApoC and ApoE from VLDL. In addition, phospholipid transfer protein (PLTP) promotes the transfer of phospholipids from VLDL to HDL. During this process, HDL becomes enriched in triacylglycerols and phospholipids that are both degraded by hepatic lipase, thus forming smaller HDL particles that can be cleared by the liver or again participate in reverse cholesterol transport. During the catabolic process, lipid-poor ApoA-I is formed that can be filtered at the level of the glomerulus and then catabolised by proximal renal tubular epithelial cells after binding to cubilin, a protein localised to the apical surface of the renal tubular cell [13].

### Lipid transfer proteins

CETP and PLTP play key roles in lipoprotein metabolism. CETP facilitates the transport of cholesteryl esters and triacylglycerols between the lipoproteins, resulting in: (1) a net loss of cholesterol esters and gain of triacylglycerols by HDL and LDL; and (2) a reciprocal net gain of cholesterol esters and loss of triacylglycerols by chylomicrons and VLDL. CETP activity is increased by triacylglycerol-rich lipoproteins, NEFA and some phospholipids (phosphatidylcholine) and is inhibited by ApoC-I [14]. Any changes in CETP activity significantly modify the composition and metabolism of lipoproteins. PLTP circulates in the plasma bound to HDL, mediating the transfer of phospholipids (e.g. from VLDL remnants) into these particles, and also the exchange of phospholipids between lipoproteins.

### Adiponectin

Adiponectin is thought to play a direct role in influencing lipid metabolism. Adiponectin has been shown to be inversely correlated with fasting and postprandial triacylglycerols [15, 16]. It facilitates ApoA-I-mediated cholesterol efflux from macrophages by upregulating ABCA1 expression [17]. In addition, adiponectin is positively correlated with plasma HDL-cholesterol levels, and some data indicate that adiponectin may directly reduce HDL catabolism [18].

## The role of insulin in lipoprotein metabolism

Insulin is a key hormone in the regulation of lipid metabolism. The main sites of action of insulin on lipoprotein metabolism are shown in Fig. 1. In adipose tissue, insulin has an antilipolytic effect, inhibiting hormone-sensitive lipase. Thus, it promotes storage of triacylglycerols in adipocytes and reduces secretion of circulating NEFA from adipose tissue. This inhibition of lipolysis by insulin is particularly effective during the postprandial period.

Insulin directly inhibits hepatic VLDL production. In individuals with normal lipid metabolism, insulin has been shown to induce a 66% decrease in VLDL-triacylglycerol production and a 53% decrease in VLDL-ApoB production [19]. Insulin reduces VLDL production by diminishing circulating levels of NEFA, which are substrates for VLDL, and by exerting a direct inhibitory effect on VLDL production in hepatocytes [20]. There are several findings indicating that the phosphatidylinositol 3-kinase (PI3K) pathway is involved in the inhibitory effect of insulin on VLDL secretion. The binding of insulin to its receptor induces tyrosine phosphorylation of insulin receptor substrates leading to activation of PI3K, which, once activated, induces the transformation of phosphatidylinositol 4,5-bisphosphate (PIP2) into phosphatidylinositol 3,4,5-trisphosphate (PIP3), leading to promotion of the activation of Akt, a serine/threonine kinase implicated as an effector of metabolic actions of insulin. Indeed, insulin has been shown to inhibit, via PI3K, the maturation phase of VLDL assembly by preventing bulk lipid transfer to VLDL precursors [21, 22]. This effect could be partly explained by the inhibition by insulin of phospholipase D1 and ARF-1, two factors involved in the formation of VLDL_1_ [6, 10]. In addition, insulin activation promotes the PERPP of ApoB via PI3K [23]. It has also been shown that insulin reduces the synthesis of ApoB by inhibiting *ApoB* mRNA translation [24]. Furthermore, insulin negatively regulates *MTP* (also known as *MTTP*) gene expression [10]. Insulin is a potent activator of LPL, promoting the catabolism of triacylglycerol-rich lipoproteins (e.g. chylomicrons, VLDL) [15]. Insulin also inhibits the expression of ApoC-III, an inhibitor of LPL [25]. In addition, it induces the translocation of LRP to the plasma membrane, increasing the uptake and clearance of chylomicron remnants [26].

Insulin also promotes the clearance of LDL by increasing LDL-receptor expression and activity [27]. Some data indicate that insulin may stimulate the activity of hepatic lipase [28], but hepatic lipase responsiveness to insulin is still controversial [29]. A study on individuals without diabetes reported that insulin infusion did not exert any significant effects on LCAT or CETP activity [30].

## Lipid abnormalities in type 2 diabetes

**Fig. 3:**
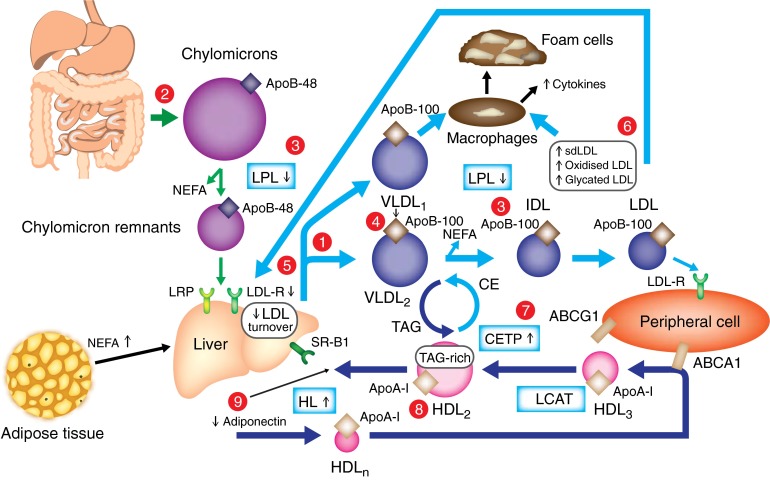
Main lipid abnormalities in type 2 diabetes. **Triacylglycerols (hypertriglyceridaemia, qualitative and kinetic abnormalities):** (1) increased VLDL production (mostly VLDL_1_), (2) increased chylomicron production, (3) reduced catabolism of both chylomicrons and VLDLs (diminished LPL activity), (4) increased production of large VLDL (VLDL_1_), preferentially taken up by macrophages; **LDL (qualitative and kinetic abnormalities):** (5) reduced LDL turnover (decreased LDL B/E receptor), (6) increased number of glycated LDLs, small, dense LDLs (TAG-rich) and oxidised LDLs, which are preferentially taken up by macrophages; **HDL (low HDL-cholesterol, qualitative and kinetic abnormalities):** (7) increased CETP activity (increased transfer of triacylglycerols from TAG-rich lipoproteins to LDLs and HDLs), (8) increased TAG content of HDLs, promoting HL activity and HDL catabolism, (9) low plasma adiponectin favouring the increase in HDL catabolism. CE, cholesterol esters; CETP, cholesteryl ester transfer protein; dLDL, small, dense LDL; HDL_n_, nascent HDL; HL, hepatic lipase; HSL, hormone-sensitive lipase; LPL, lipoprotein lipase; sLDL-R, LDL receptor; SR-B1, scavenger receptor B1; TAG, triacylglycerol

Dyslipidaemia in individuals with type 2 diabetes is very common, with a prevalence of 72–85% [3, 31]. This phenomenon is associated with a significantly increased risk of coronary artery disease relative to individuals without diabetes [3]. Lipid abnormalities observed in patients with type 2 diabetes play a central role in the development of atherosclerosis. These lipid abnormalities are not only quantitative, but also qualitative and kinetic in nature [6–8]. Increased triacylglycerols and reduced HDL-cholesterol are the main quantitative lipid abnormalities of diabetic dyslipidaemia. In addition, patients with type 2 diabetes show qualitative and kinetic abnormalities for all lipoproteins (see Text box) [6–8, 32]. All of these abnormalities are known to be risk factors for the development of atherosclerosis [33]. The main lipid abnormalities observed in type 2 diabetes are shown in Fig. 3.

**Table:** 

**Key changes in lipoprotein metabolism in type 2 diabetes**			
Lipoprotein	Quantitative changes	Qualitative changes	Kinetic/metabolic changes
Chylomicron	• Increased plasma concentration	• Very few data (decreased ApoE content in diabetic rabbits)	• Increased production • Decreased catabolism
VLDL	• Increased plasma concentration	• Greater proportion of larger particles (VLDL_1_) • Increased palmitic acid-containing species and diacylglycerol, reduced sphingomyelin • Glycation	• Increased production • Decreased catabolism
LDL	• No change or slightly increased plasma concentration	• Greater proportion of small, dense particles (triacylglycerol enrichment) • Increased LDL oxidation • Increased palmitic acid-containing species and diacylglycerol, reduced sphingomyelin • Glycation	• Decreased catabolism
HDL	• Decreased plasma concentration	• Triacylglycerol enrichment • Reduced phospholipids, ApoE and ApoM • Glycation	• Increased catabolism

It is important to note that many of the lipid abnormalities observed in patients with type 2 diabetes exist before the onset of diabetes as part of the insulin-resistant metabolic syndrome which is characterised by the accumulation of triacylglycerol-rich lipoproteins and small, dense LDL particles with reduced HDL-cholesterol in plasma. These lipid abnormalities are known to promote cardiovascular disease in individuals with the metabolic syndrome [34]. This emphasises the important role of insulin resistance in the pathophysiology of diabetic dyslipidaemia, which is also highlighted by the presence of lipid abnormalities typical of diabetic dyslipidaemia in non-diabetic insulin-resistant first-degree relatives of patients with type 2 diabetes [35, 36].

### Cholesterol absorption and synthesis

Patients with type 2 diabetes have a reduced plasma level of campesterol, a marker of cholesterol absorption, and increased plasma levels of lathosterol, a marker of cholesterol synthesis [37]. Using peroral administration of isotopes, reduced cholesterol absorption and increased cholesterol synthesis have been demonstrated in patients with type 2 diabetes [38]. The mechanisms responsible for these changes in cholesterol homeostasis are not yet clarified. In a study performed in 263 patients with type 2 diabetes, liver fat content was independently associated with plasma lathosterol [39]. It has been suggested that this could be due to increased expression of *SREBP*2, encoding sterol regulatory element-binding protein, a factor regulating cholesterol uptake and synthesis, observed under conditions of increased liver fat content [40].

### Postprandial hyperlipidaemia and chylomicrons

In individuals with type 2 diabetes and insulin resistance, an increase in chylomicron production is observed, contributing to the postprandial hyperlipidaemia observed in this population [41]. Indeed, patients with type 2 diabetes have an increased rate of intestinal ApoB-48 secretion [42] and augmented expression of *MTP* (responsible for the addition of triacylglycerols to ApoB-48) within the intestine [43]. Insulin resistance is likely to be involved in the increased chylomicron production, since the normal acute suppression of postprandial chylomicron secretion, by insulin, is absent in patients with type 2 diabetes [44]. In addition, increased plasma NEFA concentrations (as a result of reduced inhibition of hormone-sensitive lipase in type 2 diabetes [45]) may further drive ApoB-48 secretion [46]. The clearance of chylomicrons is also impaired in type 2 diabetes [47]. Several mechanisms are responsible for this delayed chylomicron catabolism. The activity of LPL, the enzyme responsible for chylomicron hydrolysis, is significantly reduced in patients with type 2 diabetes [15, 48]. Insulin resistance is also associated with increased plasma levels of ApoC-III, an inhibitor of LPL [49]. Furthermore, the activation of LRP (one of the hepatic receptors responsible for chylomicron remnant uptake) by insulin is abolished in insulin-resistant mice [26]. The net result of all these changes is a significantly larger pool of chylomicrons (hypertriglyceridaemia) (Fig. 3). Moreover, patients with type 2 diabetes show increased levels of atherogenic remnant particles, including chylomicron remnants and VLDL remnants [50].

Postprandial hyperlipidaemia is likely to promote atherosclerosis and the occurrence of cardiovascular events in patients with type 2 diabetes. The increase in postprandial triacylglycerols has been shown to be correlated with the increase in TNFα, IL-6 and vascular cell adhesion molecule 1 (VCAM-1) values, in patients with type 2 diabetes, indicating a deleterious proinflammatory effect of postprandial hyperlipidaemia [51]. Furthermore, the magnitude of postprandial hypertriglyceridaemia is strongly correlated with the reduction in flow mediated dilatation, in patients with type 2 diabetes, indicating a role for postprandial hyperlipidaemia in endothelial dysfunction [52].

### VLDLs and IDLs

Increased plasma triacylglycerol levels in patients with type 2 diabetes are largely due to an increased number of VLDLs, particularly large VLDL_1_ particles [6]. Both increased production and delayed catabolism of VLDL are responsible for the increased VLDL pool. In vivo kinetic studies in patients with type 2 diabetes have shown an augmented production of both VLDL-ApoB and VLDL-triacylglycerols [53–55]. More precisely, it has been demonstrated that type 2 diabetes is associated with increased production of large VLDL_1_ particles [56, 57]. Similar increases in VLDL production have been observed in obese, non-diabetic, insulin-resistant individuals, suggesting a critical role of insulin resistance in the pathophysiology of VLDL overproduction in type 2 diabetes [58, 59]. It has been shown that the VLDL_1_ production rate is correlated with insulin resistance and liver fat in patients with type 2 diabetes [57, 60].

**Fig. 4:**
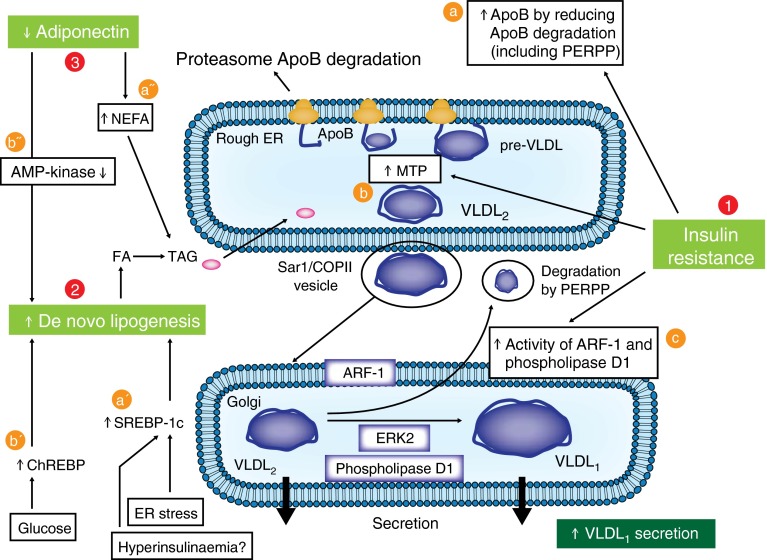
Pathophysiology of increased hepatic VLDL production in type 2 diabetes. 1. Insulin resistance is responsible for: (a) a reduction in ApoB degradation, leading to an increased ApoB level in hepatocytes (including ApoB degradation by PERPP); (b) increased MTP expression; and (c) increased activity of two factors involved in the formation of VLDL_1_, phospholipase D1 and ARF-1. Moreover, peripheral insulin resistance is responsible for increased levels of NEFA, which activate VLDL production (see a′′). 2. Increased de novo lipogenesis secondary to: (a′) increased activation of SREBP-1c (by ER stress); and (b′) increased activation ChREBP (by hyperglycaemia). 3. Reduced plasma adiponectin level responsible for: (a′′) increased plasma NEFA levels as a consequence of reduced muscle NEFA oxidation; and (b′′) a reduction in AMP-kinase activation in the liver, which promotes de novo lipogenesis. COPII, coat protein II; ERK2, extracellular signal-regulated kinase 2; FA, fatty acid; TAG, triacylglycerol;

Insulin resistance is associated with reduced inhibition of hormone-sensitive lipase in adipose tissue by insulin, leading to increased lipolysis and, thereby, augmented NEFA portal flux to the liver. This has been shown to stimulate synthesis of triacylglycerols in hepatocytes [10]. Furthermore, the normal suppressant effect of insulin on postprandial VLDL (more specifically, VLDL_1_) production is blunted by hepatic insulin resistance [45, 56, 61]. Several mechanisms seem to be involved in the overproduction of hepatic VLDL relative to the reduced inhibitory effect of insulin (Fig. 4). First, data from animal studies have provided evidence that insulin resistance is associated with a reduction in ApoB degradation in hepatocytes, leading to an increase in the ApoB pool available for VLDL assembly [62, 63]. Reduced PI3K activity in animal models, secondary to insulin resistance, has been reported to increase the expression of protein-tyrosine phosphatase 1B (PTP-1B), leading to suppression of ER60, a protease associated with the ER, which promotes ApoB degradation via a non-proteasomal pathway [62, 64]. In addition, an increased NEFA level in hepatocytes reduces the post-translational degradation of ApoB. Second, *MTP* expression is increased in insulin-resistant states and type 2 diabetes [65]. In insulin resistance, the reduced activation of PI3K leads to increased forkhead box protein O1 (FOXO1) activation, which is normally inhibited by activated PI3K. This increased activation of FOXO1 is responsible for augmented transcription of the *MTP* gene [66]. Third, it has been suggested that insulin resistance could be responsible for the increased activity of two factors involved in the formation of VLDL_1_: phospholipase D1 and ARF-1 [21]. It has also been suggested that decreased PIP3, secondary to reduced PI3K activation, may also be involved, since PIP3, a highly negatively charged phospholipid, reduces lipid transfer to VLDL precursor and, thus, the formation of VLDL_1_ [22]. It is possible that the increased formation of VLDL_1_ automatically reduces the amount of VLDL precursors available for PERPP and, thus, decreases the rate of ApoB degradation [22].

In addition, de novo lipogenesis is increased in individuals with insulin resistance [67]. This increased de novo lipogenesis is secondary to augmented expression of both carbohydrate responsiveness element-binding protein (ChREBP) and sterol regulatory element-binding protein (SREBP)-1c in insulin resistance and type 2 diabetes [10]. It is suspected that, in patients with type 2 diabetes, hyperglycaemia directly activates ChREBP [68]. The increase in SREBP-1c expression could be related to the augmented ER stress observed in insulin resistance and type 2 diabetes [69]. Based on animal studies, it has been proposed that hyperinsulinaemia observed in insulin resistance and type 2 diabetes might be responsible for increased SREBP-1c expression because insulin stimulates SREBP-1c transcription. However, this is not supported by data in humans, since hyperinsulinaemia in patients with insulinoma is not associated with increased VLDL production [70]. Furthermore, insulin treatment in patients with type 2 diabetes induces a reduction in liver fat rather than an increase [71]. Moreover, reduced plasma adiponectin levels observed in type 2 diabetes may also promote VLDL production by increasing plasma NEFA levels, as a consequence of reduced muscle NEFA oxidation, and by inducing a decrease in AMP-kinase activation in the liver, which promotes de novo lipogenesis [10].

As assessed by kinetic studies using radioisotopes [54] and stable isotopes [53], catabolism of VLDLs is reduced in patients with type 2 diabetes, which also promotes hypertriglyceridaemia. This defect in VLDL catabolism mainly reflects the reduced activity of LPL in type 2 diabetes, particularly in adipose tissue [48]. Since insulin is an activator of LPL, it has been suggested that the diminution of LPL activity may be due to a relative insulin deficiency and/or insulin resistance. In addition, increased plasma levels of ApoC-III (an inhibitor of LPL) could also contribute to the decreased catabolism of VLDL in patients with type 2 diabetes, since increased plasma levels of ApoC-III were associated with impaired VLDL clearance in obese insulin-resistant men [72]. Moreover, postprandially, chylomicrons and VLDL compete for LPL, which exacerbates postprandial hypertriglyceridaemia. Most of the time, hypertriglyceridaemia observed in patients with type 2 diabetes is mild to moderate but the severity of hypertriglyceridaemia is also influenced, in each patient, by the genetic susceptibility [73]. In some sporadic cases of high genetic susceptibility, severe hypertriglyceridaemia may develop, with attendant risk of pancreatitis, particularly in hyperglycaemia [73].

The type of VLDL particles produced in type 2 diabetes is also altered, with a shift towards a greater proportion of those of a larger particle size (VLDL_1_) [32, 74]. Relative to smaller VLDL particles, these are enriched with cholesterol esters and phospholipids. Larger triacylglycerol-enriched VLDL particles are potentially more atherogenic, as indicated by their significant association with endothelial dysfunction [52], and their preferential uptake by macrophages, leading to the formation of foam cells in vessel walls [75]. It has recently been demonstrated that VLDL from patients with type 2 diabetes has increased diacylglycerol content and reduced sphingomyelin, as well as increased palmitic acid-containing species [76]. Since palmitate is the major fatty acid synthesised during de novo lipogenesis, the increased palmitic acid content in VLDL-triacylglycerols might be a consequence of increased de novo lipogenesis, as suggested by a recent study that reported increased levels of circulating triacylglycerol-containing palmitic acid in insulin-resistant obese individuals with non-alcoholic fatty liver disease [77]. In addition, increased palmitic acid in VLDL-triacylglycerols has been shown to promote secretion of proinflammatory mediators by human smooth muscle cells [76]. Glycation of apolipoproteins in VLDL (ApoB, ApoCs, ApoE) may occur in diabetes. This may reduce VLDL binding to the ApoB/E receptor and hence impair its catabolism [78]. Furthermore, it has been suggested that glycation of ApoC-II, a cofactor of LPL, could contribute to lesser LPL activation [79].

### LDLs

In patients with type 2 diabetes, the mean LDL-cholesterol level is comparable or slightly elevated relative to that in individuals without diabetes [6, 8, 32]. However, the catabolism of LDL is substantially reduced [53, 80], inducing a longer duration of LDL in plasma that may promote lipid deposition within artery walls. In patients with type 2 diabetes, the number of LDL B/E cell-surface receptors is significantly reduced, which may be due to reduced insulin-mediated expression and could be responsible for observed impairments in LDL catabolism [81]. It has also been suggested that reduced LDL catabolism could be partly attributed to a decreased affinity of LDL for its receptor following ApoB glycation [82].

As a consequence of hyperglycaemia, increased glycation of LDL is observed in individuals with type 2 diabetes [83]. Glycated LDL has reduced affinity for LDL B/E receptors [84] and is preferentially taken up by macrophages, leading to the formation of foam cells [85]. Another lipoprotein modification observed in type 2 diabetes that has marked atherogenic potential is increased LDL oxidation. Patients with type 2 diabetes show increased oxidisability of LDLs and have an augmented number of oxidised LDL particles in their plasma [8]. Oxidised LDLs demonstrate a decreased affinity for the LDL receptor, and are preferentially taken into macrophages via specific oxidised LDL receptors prior to foam cell development [86]. In addition, they have chemoattractant effects on monocytes by increasing the formation of adhesion molecules, such as intercellular adhesion molecule 1 (ICAM-1), by endothelial cells, and by stimulating the formation of cytokines, such as TNFα or IL-1, by macrophages, which amplifies the inflammatory atherosclerotic process [86].

Small, dense, triacylglycerol-rich LDL particles (known as subclass B particles) are more prevalent in type 2 diabetes [87]. This is mainly related to hypertriglyceridaemia, and VLDL_1_ triacylglycerol is the major predictor of LDL size in patients with type 2 diabetes and in non-diabetic individuals [6]. The characteristic hypertriglyceridaemia observed in patients with type 2 diabetes stimulates CETP, leading to the preferential formation of triacylglycerol-rich small, dense LDL particles over larger ones [74]. The presence of small, dense LDL particles has been reported to be associated with increased cardiovascular risk and progression of atherosclerosis [88]. Small, dense LDL particles are more atherogenic. They are more likely to undergo glycation and oxidation than larger LDL particles, which promotes the generation of foam cells [8, 89]. In addition, they show increased affinity for intimal proteoglycans, which may favour the penetration of LDLs into the arterial wall [90]. Individuals with small, dense LDL particles have an impaired response to the endothelium-dependent vasodilator acetylcholine [91]. Furthermore, as with VLDL, LDL from patients with type 2 diabetes shows significant changes in both lipid class (increased diacylglycerol and reduced sphingomyelin) and lipid species (increased palmitic acid-containing species), and the level of palmitic acid in LDL is correlated with insulin resistance [76].

### HDLs

Plasma levels of HDL-cholesterol and ApoA-I are reduced in patients with type 2 diabetes [6–8]. In particular, the proportion of circulating smaller HDL particles (HDL_3_) is increased, while there are far fewer large HDL particles (HDL_2_); therefore, the overall number of HDL particles is reduced [74]. Reduced levels of HDL_2_ in patients with type 2 diabetes have been reported to be associated with both hypertriglyceridaemia and obesity [92]. Kinetic studies using radioisotopes [93] and stable isotopes [94] have demonstrated that the decrease in HDL-cholesterol in patients with type 2 diabetes is due to increased catabolism of HDLs. The activity of hepatic lipase, the enzyme controlling HDL catabolism, is augmented in insulin-resistant states, which is likely to be responsible for the observed increase in HDL catabolism [95]. Hypertriglyceridaemia is a major contributing factor to the accelerated HDL catabolism observed in type 2 diabetes. It has recently been demonstrated that both increased VLDL_1_ production and reduced VLDL_1_ catabolism are independent factors associated with increased HDL catabolism in insulin-resistant states [96]. It is suggested that the increased pool of triacylglycerol-rich lipoproteins (mainly VLDL_1_), observed in type 2 diabetes, promotes CETP-mediated triacylglycerol enrichment of HDL particles and, as a consequence, enhances HDL catabolism, since HDL-rich particles are very good substrates for hepatic lipase. The reduction in plasma adiponectin levels observed in individuals with insulin resistance and type 2 diabetes may be another mechanism involved in the diminution of HDL-cholesterol levels. Indeed, a significant negative correlation has been reported between the rate of HDL-ApoA-I catabolism and plasma levels of adiponectin, independently of abdominal obesity, insulin sensitivity, age, sex and plasma lipids, suggesting a direct effect of adiponectin on HDL metabolism [18]; however, its precise role has yet to be determined.

Several qualitative abnormalities in HDLs have been described in patients with type 2 diabetes. They are enriched in triacylglycerols, and this enrichment is responsible for increased HDL catabolism, as previously discussed. Furthermore, HDLs are glycated in type 2 diabetes, although the exact consequences of this glycation remain unknown. The reduction in phospholipids in large HDL particles in patients with type 2 diabetes is associated with increased arterial stiffness [97]. Patients with type 2 diabetes also have reduced ApoE content in large HDL particles, which may have an atherogenic effect, since large, ApoE-rich HDL usually prevents LDL binding to proteoglycans in the vessel wall [97]. ApoM, which is mainly associated with HDL, is reduced in patients with type 2 diabetes due to diabetes-associated obesity [97, 98]. ApoM promotes the formation of pre–β-HDL from α-HDL, and lower levels of ApoM may explain why pre-β-HDL formation is not increased in type 2 diabetes, despite increased PLTP activity. ApoM mediates the enrichment in sphingosine-1-phosphate in HDL, which promotes arterial vasodilation by stimulating endothelial nitric oxide formation [99].

In patients with type 2 diabetes, HDL has a reduced capacity to promote ex vivo cholesterol efflux from cells. This may be due to reduced expression of ABCA1, which is the membrane transporter responsible for the first step of cholesterol transfer from cell membranes to HDL [100]. Furthermore, glycation of ABCA1 has been shown to reduce its activity [101]. In addition, expression of ABCG1, another transporter involved in reverse cholesterol transport, is reduced in monocytes from patients with type 2 diabetes, and this is associated with impaired cholesterol efflux [102].

Reductions in the antioxidative effects of HDLs, promoted by hyperglycaemia and triacylglycerol enrichment, have been reported in patients with type 2 diabetes [103]. Furthermore, the ability of HDL to counteract the inhibition of endothelium-dependent vasorelaxation induced by oxidised LDL is impaired in type 2 diabetes. This reduction in HDL vasorelaxant effects is inversely correlated with HDL triacylglycerol content [104]. In line with these data, HDL from patients with type 2 diabetes has a weaker stimulatory effect on endothelial nitric oxide synthesis [105].

### Lipid transfer proteins

The qualitative lipoprotein abnormalities observed in patients with type 2 diabetes, such as increased triacylglycerol content of LDL and HDL particles, indicate increased CETP activity [106]. The main factor responsible for the increased CETP activity in type 2 diabetes is the augmented pool of triacylglycerol-rich lipoproteins (mainly VLDL), which directly stimulate CETP. However, hyperglycaemia per se could also activate CETP, since glycation of lipoproteins increases CETP activity [107]. In addition, a recent study in patients with diabetes reported that glycation of ApoC-I reduces its inhibitory effect on CETP [108]. Increased PLTP mass and PLTP activity have also been reported in patients with type 2 diabetes [109], and this is associated with increased intima–media thickness [110].

### A putative role of some adipocytokines and proteins in the pathophysiology of diabetic dyslipidaemia?

#### Adiponectin

Adiponectin has a generally anti-atherogenic profile that may be partly due to its beneficial action on lipid metabolism [111]. However, adiponectin levels are reduced in patients with type 2 diabetes, and so its cardioprotective effects are minimised.

In non-diabetic individuals, as in patients with type 2 diabetes, plasma adiponectin is negatively correlated with plasma triacylglycerols and positively correlated with plasma HDL-cholesterol, and these associations are independent of insulin resistance [16, 112]. Low adiponectin plasma levels are associated with augmented VLDL catabolism [113] and coupled with reduced LPL activity in adipose tissue [114] independently of insulin resistance, suggesting a possible direct action of adiponectin on lipid metabolism, independent of its effects on insulin sensitivity. Adiponectin may decrease plasma triacylglycerols by enhancing NEFA oxidation or by stimulating lipoprotein lipase [111, 115]. In addition, a significant negative correlation has been reported between HDL-ApoA-I catabolism and plasma adiponectin, independent of insulin resistance and plasma lipids, suggesting a direct effect of adiponectin on HDL metabolism [18]. However, the exact mechanisms that may explain a direct effect of adiponectin on VLDL and HDL have not yet been clarified.

#### Retinol-binding protein 4

Levels of retinol-binding protein 4 (RBP4), an adipokine secreted by adipocytes and the liver, are elevated in type 2 diabetes. An independent association between RBP4 and triacylglycerols has been reported [116, 117]. Moreover, a strong, independent, negative association has been reported between plasma RBP4 and VLDL catabolism in patients with type 2 diabetes, suggesting that RBP4 may be involved in the pathophysiology of hypertriglyceridaemia in type 2 diabetes [117]. Further studies are needed to clarify the potential role of RBP4 in diabetic dyslipidaemia.

## Conclusions

Abnormalities of lipoprotein metabolism are one of the major factors contributing to cardiovascular risk in patients with type 2 diabetes, and diabetic dyslipidaemia includes not only quantitative but also qualitative and kinetic lipoprotein abnormalities that are inherently atherogenic. The primary (characteristic) quantitative abnormalities are hypertriglyceridaemia, accompanied by prolonged postprandial hyperlipidaemia and increased levels of remnant particles (related to the increased production of triacylglycerol-rich lipoproteins and a reduction in the rate of catabolism of triacylglycerol-rich lipoproteins), and decreased HDL-cholesterol levels secondary to an increased rate of HDL catabolism. The most frequent qualitative abnormalities, which are potentially atherogenic, include an increase in large VLDL particle size (VLDL_1_); a greater proportion of small, dense LDL particles; an augmented susceptibility of LDLs to oxidation; an increase in triacylglycerol content of both LDL and HDL; and glycation of apolipoproteins. Although levels of LDL may be normal in patients with type 2 diabetes, LDL plasma residence time is increased due to a slower turnover rate, and this may infer the promotion of lipid deposition within artery walls. Furthermore, the usual cardioprotective effects of HDL are reduced or abolished in type 2 diabetes. Some factors, such as insulin resistance and possibly some adipokines (e.g. adiponectin) and hyperglycaemia, are involved in the pathophysiology of diabetic dyslipidaemia. However, many questions remain unanswered (such as the pathophysiology and the consequences of the qualitative lipid abnormalities, the precise mechanisms and signalling pathways involved in the insulin resistance linked lipid abnormalities, the potential role of adipose tissue and adipocytokines in the pathophysiology of diabetic dyslipidaemia) and additional studies are needed to gain further insight into the precise mechanisms of diabetic dyslipidaemia. Deeper understanding of lipid disorders in type 2 diabetes should lead to better treatment of diabetic dyslipidaemia.

## Abbreviations

ABCA1: ATP-binding cassette protein 1
ABCG1: ATP-binding cassette G1
Apo: Apolipoprotein
ARF-1: ADP ribosylation factor 1
CETP: Cholesteryl ester transfer protein
ChREBP: Carbohydrate responsive element-binding protein
ER: Endoplasmic reticulum
FOXO1: Forkhead box protein O1
HMG-CoA: 3-Hydroxy-3-methylglutaryl coenzyme A
LCAT: Lecithin–cholesterol acyltransferase
ICAM-1: Intercellular adhesion molecule 1
IDL: Intermediate-density lipoprotein
LPL: Lipoprotein lipase
LRP: LDL receptor–related protein
MTP: Microsomal triacylglycerol transfer protein
PERPP: Post-ER presecretory proteolysis
PI3K: Phosphatidylinositol 3-kinase
PIP2: Phosphatidylinositol 4,5-bisphosphate
PIP3: Phosphatidylinositol 3,4,5-trisphosphate
PLTP: Phospholipid transfer protein
RBP4: Retinol-binding protein 4
PTP-1B: Protein-tyrosine phosphatase 1B
SREBP: Sterol regulatory element-binding protein
